# Incidence and surgical care of retinal detachment during the first SARS-CoV-2 lockdown period at a tertiary referral center in Austria

**DOI:** 10.1371/journal.pone.0248010

**Published:** 2021-03-08

**Authors:** Markus Schranz, Michael Georgopoulos, Stefan Sacu, Adrian Reumueller, Gregor S. Reiter, Georgios Mylonas, Ursula Schmidt-Erfurth, Andreas Pollreisz

**Affiliations:** 1 Department of Ophthalmology and Optometry, Medical University of Vienna, Vienna, Austria; 2 OPTIMA, Christian Doppler Laboratory, Department of Ophthalmology and Optometry, Medical University of Vienna, Vienna, Austria; Medizinische Universitat Graz, AUSTRIA

## Abstract

**Purpose:**

To assess the influence of the SARS-CoV-2 lockdown in spring on frequency, severity and quality of care of rhegmatogeneous retinal detachments (RRD) in a tertiary referral center in Vienna, Austria.

**Methods:**

Single center, consecutive case series with historical controls. Patients presenting with primary RRD during the first Austrian SARS-CoV-2 lockdown (March 16^th^–May 3^rd^ 2020) and a corresponding control group consisting of the same time period of the preceding 3 years.

**Results:**

The mean number of patients with RD in the reference group (RG) was 22 **(± 1)** and in the lockdown group (LG) 15. Median total delay, defined as onset of symptoms until surgery, in the RG was 5 (lower quartile: 3.0; upper quartile: 8.0) compared to 7 (3.0; 12.0) days in the LG, (p = 0.740). During the lockdown 67% of patients were referred from an external ophthalmologist compared to 52% in the RG, (p = 0.395). 34% of patients in the RG presented with an attached macula compared to 33% in the LG (p = 0.597). PVR was present in 49% of cases in the RG compared to 73% in the LG. Single surgery success (SSS) rates were lower in the LG (73.3%) compared to the RG (85.3%), (p = 0.275).

**Conclusion:**

Patients with RRD during the SARS-CoV-2 lockdown presented and were treated within acceptable time limits, showed the same macula-on ratios but a higher PVR rate and a tendency towards worse SSS rates compared to the time period of the preceding 3 years.

## Introduction

At the end of the year 2019 a new disease appeared in the Chinese city of Wuhan, in the province of Hubai. It was quickly known as the coronavirus disease 2019 (COVID-19), caused by the newly discovered virus called severe acute respiratory syndrome coronavirus 2 (SARS-CoV-2) [1]. Due to the high virulence of the virus, its basic reproduction number (R0-value) was estimated between 2.24 and 3.58 [1], the disease spread quickly over Asia, Europe and Northern America. With more than 20000 cases across Europe at the 11^th^ of March 2020 the World Health Organization (WHO) announced the COVID-19 outbreak a pandemic.

Countries were instructed to quickly implement or continue containment strategies to slow down the spreading of the disease. To mitigate transmission of the virus the Austrian government ordered social distancing, closure of restaurants, sport facilities, shops and malls by March 15^th^ 2020. These regulations also affected the health sector. Access restrictions especially for outpatients in hospitals were established, eligible surgeries were postponed, and only emergencies treated.

In ophthalmology, retinal detachment (RD) is considered an emergency case, which requires timely treatment to secure or rather restore visual acuity. International guidelines, that are in place at our department, recommend surgery within 7 days for macula-off RD and a within 24hrs approach for macula-on RD [2–6].

During the shutdown ordered by the federal government, we adapted our surgery schedule to the COVID-19 pandemic guidelines, released by the American Society of Retina Specialists (ASRS) and the American Academy of Ophthalmology (AAO). These guidelines define the causes for essential visits and categorize surgery indications into 3 groups with corresponding intervention timeframes: The highest priority has the “Emergent surgical indications” group, which includes RDs with macula attached and RDs with macula detached in a monocular patient. This is followed by urgent surgical indications, such as RD with macula detached or vitreous hemorrhage in which a retinal tear is suspected. The third group is defined as non-urgent, non-elective and contains indications like macular hole or diabetic vitreous hemorrhage with no macular threatening RD [7].

The aim of this retrospective study was to evaluate frequency, severity and quality of care of RD cases at the Department of Ophthalmology at the Medical University Vienna in the period of the corona crisis, March 16^th^ until May 6^th^ and to compare the results with the same time period of the preceding 3 years.

## Methods

This retrospective study was conducted at the Department of Ophthalmology of the Medical University of Vienna (MUV, Vienna, Austria). The study was approved by the local ethics committee (Ethics Committee of the Medical University of Vienna) and adhered to the tenets of the Declaration of Helsinki.

We collected fully anonymized data of patients diagnosed with RD at the Department of Ophthalmology at MUV during the SARS-CoV-2 lockdown period in Austria covering the time period from the 16^th^ of March 2020 to the 3^rd^ of May 2020 and compared them with data from the same time period of the preceding 3 years (20t^th^ of March - 7^th^ of May 2017, 19^th^ of March– 6^th^ of May 2018, 18^th^ of March– 5^th^ of May 2019).

Inclusion criteria were rhegmatogeneous RD, first occurrence and a healthy fellow eye.

Exclusion criteria were tractional RD, exudative RD, trauma, and RD in the fellow eye.

The surgeons and physicians were the same over the investigated time periods.

The following data were obtained from the patients (Table 1): gender, age, date of first presentation, route of referral (presentation at emergency unit, referral by ophthalmologist, referral by another ophthalmology clinic, incidental finding during checkup at our department), laterality, visual acuity (VA); lens status (phakic and clear, cataract, pseudophakia, sulcus fixated intraocular lens, anterior chamber lens or aphakia.

**Table 1:** Results, normal distributed data represented by the mean ± standard deviation, not normal distributed data represented by the median (lower quartile; upper quartile).

	Time period	Time period	p- value
	**2017–2019**	**2020**	
	March 20^th^–May 7^th^ 2017, March 19^th^–May 6^th^ 2018, March 18^th^–May 5^th^ 2019	March 16^th^—May 3^rd^	
**General Data**			
**Patient number**	**22.3 (± 1.2)**	**15**	
	**2017: 21**		
	**2018: 23**		
	**2019: 23**		
**Age (years)**	**58.7 ± 9.9**	**63.1 ± 13.0**	*0*.*144*
**Gender**	**Male: 13.3 ±5.5 (60%)**	**Male: 10 (67%)**	*0*.*617*
	**Female: 9 ±4.6 (40%)**	**Female: 5 (33%)**	
**Lens status (prior to surgery)**	**Phakic: 13.7 ±1.5 (61.4%)**	**Phakic: 8 (53%)**	*0*.*318*
	**Pseudophakic: 8.3 ±2.5 (37.2%)**	**Pseudophakic: 6 (40%)**	
	**Aphakic: 0.3 ±0.6 (1.4%)**	**Aphakic: 0**	
	**Secondary IOL: 0**	**Secondary IOL: 1 (7%)**	
**Retinal Detachment morphology**			
**Involvement of quadrants (amount)**	**I: 5.7 ±1.5 (25.6%)**	**I: 2 (13.3%)**	*0*.*650*
	**II: 10.3 ±3.1 (46.2%)**	**II: 8 (53.3%)**	
	**III: 4 ±1.7 (17.9%)**	**III: 4 (26.7%)**	
	**IV: 2.3 ±1.5 (10.3%)**	**IV: 1 (7.7%)**	
**Holes (number)**	**2.87 ± 1.74**	**3.29 ± 2.16**	*0*.*434*
**Proliferative vitreoretinopathy status**	**None: 11.3 ±2.3 (51%)**	**None: 4 (27%)**	*0*.*191*
	**Grade A: 2.3 ±1.2 (10%)**	**Grade A: 4 (27%)**	
	**Grade B: 6.7 ±2.9 (30%)**	**Grade B: 5 (33%)**	
	**Grade C: 2 ±1.7 (9%)**	**Grade C: 2 (13%)**	
**Macula status**	**Macula ON: 7.7 ±1.2 (34%)**	**Macula ON: 5 (33%)**	*0*.*597*
	**Macula OFF: 14.7 ±1.2 (66%)**	**Macula OFF: 10 (67%)**	
**Off clinic ophthalmologist consultation**	**Yes: 12 ±2 (52%)**	**Yes: 10 (67%)**	*0*.*395*
	**No: 10.7 ±2.1 (48%)**	**No: 5 (33%)**	
**Delays**			
**Patient delay**	**Median: 4.0 (1.0; 8.0)**	**Median: 6.0 (1.0; 9.0)**	*0*.*484*
**Diagnosis—first clinic contact (referral/doctors delay) (days)**	**Median: 0 (0; 0)**	**Median: 0 (0; 0)**	*0*.*992*
**First presentation -Surgery (Surgery delay) (days)**	**Median: 1.0 (1.0; 2.0)**	**Median: 1.0 (1.0; 1.0)**	*0*.*852*
	**Macula-ON 1.0 (1.0; 1.0)**	**Macula-ON 1.0 (1.0; 1.0)**	
	**Macula-OFF (1.0; 2.0)**	**Macula-OFF (1.0; 1.0)**	
**First symptoms -Surgery (total delay) (days)**	**9.42 ± 13.14**	**8.6 ± 8.3**	*0*.*740*
	**Median: 5 (3.0; 8.0)**	**Median: 7 (3.0; 12.0)**	
**SURGERY Details**			
**Surgery duration (minutes)**	**64.8 ± 36.8**	**71.4 ± 25.2**	*0*.*514*
**Surgery type**	**Vitrectomy + SF6: 9 ± 3.5 (40%)**	**Vitrectomy + SF6: 6 (40%)**	0.614
	**V.C2F6: 8.6667 ± 1.5 (39%)**	**Vitrectomy + C2F6: 4 (27%)**	
	**V. + C3F8: 2.6667± 2.1 (12%)**	**Vitrectomy + C3F8: 4 (27%)**	
	**V. + Silicon oil: 1.3 ± 0.6 (6%)**	**Vitrectomy + Silicon oil: 1 (7%)**	
	**Buckling Surgery: 0.6667 ± 1.2 (3%)**	**Buckling Surgery: 0 (0%)**	
**Combined cataract surgery**	**Yes: 3 ± 1 (14%)**	**Yes: 3 (20%)**	0.992
	**No: 19.3 ± 2.5 (87%)**	**No: 12 (80%)**	
**Single surgery success**	**Yes: 19.3 ± 2.1 (85%)**	**Yes: 11 (73%)**	0.275
	**No: 3.3 ± 1.2 (15%)**	**No: 4 (27%)**	

Furthermore, the following RD specifics were recorded: RD type (rhegmatogeneous, tractional, tractional diabetic, following vitrectomy, recurring RD after surgery, RD due to ocular trauma); defect number, number of retinal quadrants detached; presence of proliferative vitreoretinopathy (PVR) stage (PVR-A, B, C) and the date when symptoms started.

We evaluated following delays in presentation of our patients:

The patient delay, defined as the period of time from onset of symptoms until the first medical consultation. The doctors delay, defined as the period of time from diagnosis until referral to our eye clinic. The surgery delay, defined as the period of time between first contact at our clinic and ocular surgery [8].

If a patient had a recurring RD within 3 months after the first surgery this patient was counted as a non-single surgery success.

We did not investigate BCVA after resorption of gas, due to the fact that some patients received surgery and were further followed up at the referring hospital or ophthalmologist.

IBM SPSS statistics 26.0 was used for statistical testing.

Shapiro-Wilk test, box-plots and histograms were used to test for normal distribution within the data.

In case of normally distributed data, the mean ± standard deviation was reported. Means were compared using students t-test with the significance threshold set to 0.05. In case of not normally distributed data the Mann-Whitney-U test was performed and the median (lower-, and upper quartile) were reported. Chi-square test was used to analyze nominal values.

No correction for multiple testing was applied due to the small sample size and the descriptive exploration of data in this study.

## Results

During the SARS-CoV-2-lockdown phase from March 16^th^ to May 3^rd^ 2020 15 patients presented with RD at the Department of Ophthalmology at the Medical University Vienna (lockdown group, LG). The reference group (RG) consisted of 21, 23 and 23 patients in the same period of the preceding years 2017, 2018 and 2019, respectively. The mean number of patients per yearly time period with RD in the reference group was 22.3. None of the 15 patients was tested positive for the SARS-CoV-2 virus. The mean age of patients was 58.7 ± 9.9 years in the RG and 63.1 ± 13.0 years in the LG, which did not differ significantly (p = 0.144). Of the mean 22.3 patients in the RG 13.3 were men and 9 women compared to 10 men and 5 women in the LG (p = 0.617). 37% in the RG and 40% in the LG were pseudophakic, which was also not statistically significantly different (p = 0.318).

Concerning the morphology of the RDs, involvement of quadrants, number of retinal holes, presence of PVR and the status of the macula (attached, detached) no statistically significant differences could be found (p = 0.650, p = 0.434, p = 0.191, p = 0.597, respectively).

The median patient delay was 4 days in the RG compared to 6 days in the LG (p = 0.484).

During the lockdown 67% (n = 10) of patients were referred from an external ophthalmologist and 33% (n = 5) showed up directly in our clinic. In the RG 52% (n = 12) of patients came with the referral from an external eye specialist, while 48% (n = 10) showed up without a referral. There was no significant difference between the two groups (p = 0.395).

The doctors delay amount to a median of 0 days in both the RG and LG (p = 0.992).

The median surgery delay was 1.0 (lower quartile (lq): 1.0; upper quartile (uq): 2.0) day in the RG and 1.0 (1.0; 1.0) in the LG. Again, there was no statistically significant difference (p = 0.852). During the lockdown none of the patients received surgery on the day of first presentation, whereas in the RG 22% of all patients were treated on the day of first presentation.

The median total delay, defined as onset of symptoms until surgery, in the RG was 5 days (lq: 3.0; uq: 8.0) compared to 7 (lq: 3.0; uq: 12.0) days in the LG, (p = 0.740).

Concerning the macula status we observed that in the RG 34% of patients showed up with an attached macula compared to 33% of patients in the LG, respectively (p = 0.597).

Concerning the PVR status 51% of cases in the RG showed no PVR, compared to 27% in the LG. PVR grade A was found in 10% of RG compared to 27% in LG, Grade B in 30% and 33% respectively. Grade C was the rarest in both groups with a percentage of 9% and 10%, respectively. These different findings in frequencies were not significant (p = 0.191).

Investigating surgery type and surgery duration there was not a significant difference (p = 0.614 and p = 0.514, respectively). In the RG 97% of all patients received pars plana vitrectomy (PPV) and gas tamponade, the remaining 3% underwent buckling surgery, whereas in the LG all patients underwent vitrectomy and gas filling.

The surgery was counted as success if no subsequent re-surgery was necessary within 3 months. The single surgery success rate in the RG was 85.3% compared to 73.3% in the LG, this difference was not significant (p = 0.275).

## Discussion

The aim of this retrospective study was to evaluate frequency, severity and quality of care of RD cases during the Sars-CoV-2 lockdown period at the Department of Ophthalmology of the Medical University Vienna and to compare the results to the ones obtained from the same time period of the preceding 3 years.

During the 48 days of the Sars-CoV-2 lockdown in Austria in March/April 2020 fifteen patients presented with rhegmatogeneous RD compared to a mean of 22.3 ± 1.2 patients in the same time period of the past 3 years (see Table 1).

In the investigated time period of the years 2017–2019 the mean age of patients with RD was 58.68 ±9.87 years while during the corona lockdown it was 63.09 ±12.96 years. These results are in range with previous reports by the Swedish RD register of the years 1996–1997 and a study by Price et al. (60.2 years and 55.9 years, respectively) [9, 10].

A higher number of male patients were treated in both groups, yet no significant difference was found. The higher incidence of RD in men has been reported in a number of previous studies [9–11].

The first contact with a medical provider in Austria in case of an acute eye disorder usually is a general ophthalmologist, rarely a general practitioner or optician. If an acute surgical treatment is needed the ophthalmologist refers the patient to an eye clinic. However, patients may also directly consult an eye clinic without any referral in case of an acute loss of vision. Due to the lockdown process many ophthalmologists changed or reduced their opening hours, so another important aspect of this work was to evaluate the duration until the first contact with an ophthalmologist from the beginning of first symptoms, which we called patient delay [12]. The median patient delay was 6.0 during the lock down compared to 4.0 days in the past 3 years, yet no significant difference was found between the examined time periods (p = 0.861). Meurs et al. investigated delays in presentation of RD in a non Covid-19 period and reported a median delay between first symptoms and contact to a healthcare provider of 3 days for macula-ON RD and 7 days for macula-OFF cases [8]. Another study evaluated patient delay in patients with RD in the Netherlands and reported similar results [12].

Around 67% of all patients with RD were referred by an ophthalmologist which is above the average of the past 3 years (52%), yet this was just a trend and not statistically significant (p = 0.395). Nevertheless, this indicates that the referral system was functional.

Besides the patient delay the doctor delay is an important factor in the patient care process and describes the time period from diagnosis until the referral to an eye clinic for surgical treatment. In the investigated reference time as well as during the lockdown the median delay was 0 days (p>0.992), so patients were sent to the eye clinic on the very same day as the diagnosis RD was made. From these results we deduce that the ophthalmological referral process worked sufficiently. The median surgery delay was 1.0 day both in the RG and the LG (p = *0*.*852)*. By differentiating between macula-ON and OFF cases we calculated a median delay of 1.0 day for both macula-ON and OFF eyes in the RG, with the same median delay in the LG for both groups. The results of the evaluated groups correspond to international guidelines for RD, which recommend surgery within the next 24 hours for macula-ON and 7 days for macula-OFF eyes [2–6].

The shorter waiting period for macula-OFF eyes during the lockdown can be explained by the cancellation of all elective surgeries and therefore available surgical capacities. However, none of our macula-ON patients in the LG was operated on the day of presentation due to the fact that the results of the SARS-CoV-2-PCR test took 12 hours during this time period.

Evaluating the morphology of the RD we could not find any significant differences concerning the amount of detached quadrants between the two time periods (p = 0.650). The majority of patients in both groups had 2 quadrants involved (RG: 46%; LG 52%), followed by 1 quadrant in the RG (25.6%) and 3 quadrants in the LG (26.7%). The detachment of 4 quadrants showed the lowest frequency in both groups (10.3% RG and 7.7% for the LG).

These findings are consistent with findings by Hendrikse et al., who reported that the relative amount of quadrants involved was 30.5%, 50.0%, 11.8% and 7.5%, in ascending order, respectively.

Analyzing the macula status we observed that in the RG the distribution between macula-ON and macula-OFF was 34% and 65% compared to 33% and 67% in the LG, respectively (p = 0.597). Other groups reported ratios ranging from 44.1% [12] to 39.8% [9] macula-ON RD, which are similar to our findings. However, recently Patel et al. published their work on RD during the COVID-19 pandemic in the area of Philadelphia, United States of America, and reported that 24% presented with macula-ON RD compared to almost 50% in the reference year [13].

Interestingly, despite the difference of time from onset of RD symptoms until the first consultation at an ophthalmologist being similar between RG and LG, with a median of 4 and 6 days, respectively, the rate of PVR was higher in the LG compared to RG (73% and 49%, respectively). A potential yet not confirmable explanation might be that patients unconsciously suppressed symptoms in the wake of the lockdown situation. Regarding the PVR status we found that in the RG group only the percentage of eyes with PVR grade 0 (51%) and C or worse (9%) were identical with the ones reported by Algvere et al. [9]. Different results were found for the relative amount of eyes with PVR grade A (10%) and B (30%). In the LG only the percentage of eyes graded as PVR A (27%) was similar to the results reported by Algvere et al. Interestingly, we found a higher relative frequency of C3F8-gas at the expense of C2F6 in the LG (27%) compared to the RG (12%).

No difference was found in the amount of combined cataract and vitrectomy surgeries between both groups (p = 0.992). The single surgery success rate of our cases in the RG and LG was 85% and 73%, respectively and is in range with data published by Heimann et al. from a prospective, multicenter randomized controlled trial. In PPV they reported a single surgery success-rate of 63.8% in phakic and 72% in pseudophakic patients [14]. Similar results were also reported by other groups [15, 16]. The difference in the single surgery success between the two groups, although not significant and in range with other published data, might be explained due to the higher proportion of PVR in the LG.

Limitations of this study include the retrospective data analysis and the low number of patients included.

In summary we found that the lockdown due to the SARS-CoV-2 outbreak did not have any negative influence on the timely referral process of patients with RD nor the time to surgery. However, surgery success rates were slightly lower in LG potentially due to more PVR cases.
